# Common and Rare EGFR and KRAS Mutations in a Dutch Non-Small-Cell Lung Cancer Population and Their Clinical Outcome

**DOI:** 10.1371/journal.pone.0070346

**Published:** 2013-07-29

**Authors:** Gerald S. M. A. Kerner, Ed Schuuring, Johanna Sietsma, Thijo J. N. Hiltermann, Remge M. Pieterman, Gerard P. J. de Leede, John W. G. van Putten, Jeroen Liesker, Tineke E. J. Renkema, Peter van Hengel, Inge Platteel, Wim Timens, Harry J. M. Groen, the CTMM Air Force Consortium

**Affiliations:** 1 University of Groningen, Department of Pulmonary Diseases, University Medical Center Groningen, Groningen, the Netherlands; 2 University of Groningen, Department of Pathology and Medical Biology, University Medical Center Groningen, Groningen, the Netherlands; 3 Department of Pathology, Martini Hospital, Groningen, the Netherlands; 4 Department of Pulmonary Diseases, Ommelander Hospital, Delfzijl, the Netherlands; 5 Department of Pulmonary Diseases, Bethesda Hospital, Hoogeveen, the Netherlands; 6 Department of Pulmonary Diseases, Martini Hospital, Groningen, the Netherlands; 7 Department of Pulmonary Diseases, Scheper Hospital, Emmen, the Netherlands; 8 Department of Pulmonary Diseases, Refaja Hospital, Stadskanaal, the Netherlands; 9 Department of Pulmonary Diseases, Wilhelmina Hospital, Assen, the Netherlands; University of Nebraska Medical Center, United States of America

## Abstract

**Introduction:**

In randomly assigned studies with EGFR TKI only a minor proportion of patients with NSCLC have genetically profiled biopsies. Guidelines provide evidence to perform EGFR and KRAS mutation analysis in non-squamous NSCLC. We explored tumor biopsy quality offered for mutation testing, different mutations distribution, and outcome with EGFR TKI.

**Patient and Methods:**

Clinical data from 8 regional hospitals were studied for patient and tumor characteristics, treatment and overall survival. Biopsies sent to the central laboratory were evaluated for DNA quality and subsequently analyzed for mutations in exons 18–21 of EGFR and exon 2 of KRAS by bidirectional sequence analysis.

**Results:**

Tumors from 442 subsequent patients were analyzed. For 74 patients (17%) tumors were unsuitable for mutation analysis. Thirty-eight patients (10.9%) had EGFR mutations with 79% known activating mutations. One hundred eight patients (30%) had functional KRAS mutations. The mutation spectrum was comparable to the Cosmic database. Following treatment in the first or second line with EGFR TKI median overall survival for patients with EGFR (n = 14), KRAS (n = 14) mutations and wild type EGFR/KRAS (n = 31) was not reached, 20 and 9 months, respectively.

**Conclusion:**

One out of every 6 tumor samples was inadequate for mutation analysis. Patients with EGFR activating mutations treated with EGFR-TKI have the longest survival.

## Introduction

The effect of EGFR tyrosine kinase inhibitors (TKI) in patients with non-small-cell lung cancer (NSCLC) depends on the EGFR mutation status. Therefore, selecting the adequate tumor specimen for mutational analysis is an important issue in making treatment decisions in NSCLC. In previous randomized studies comparing EGFR TKI therapy to regular chemotherapy, the proportion of patients with adequate tumor tissue for analysis ranged from 10 to 38% [1], [2], [3], [4], [5], [6]. Most randomized studies used different EGFR mutation tests that only examined a very limited number of hotspot mutations such as L858R and exon 19 deletions [2], [7], [8], [9], [10]. What happened with less frequent mutations is not always obvious. As EGFR mutations are only present in non-squamous NSCLC [11], accurate histological phenotyping is mandatory in order to make decisions on the type of chemotherapy and for predicting the a priori presence of mutations. The IASLC/ATS/ERS guideline recommends mutational testing in non-squamous NSCLC [12].

In Caucasian patients with non-squamous cell lung carcinoma, the KRAS mutation is most common (20–30% of cases) [13], [14], followed in frequency by mutations in the EGFR gene (10–20% of cases) [13], [15]. Within histological phenotypes, certain features appear to be associated with specific mutations, for example the micropapillary aspect of adenocarcinoma with BRAF V600 mutations [16]. Although it is advantageous for patients with activating EGFR mutations to receive EGFR TKI [2], [3], [8], [17], [18], [19], in patients with other types of genetic aberrations this treatment is not effective. For example, in a study on patients with EML4-ALK translocations a lack of tumor response to EGFR TKI was reported [20]. However, for NSCLC patients with KRAS mutations the evidence is inconclusive. Several studies showed a complete lack of response to treatment with an EGFR TKI [17], [21], [22], one study demonstrated that NSCLC patients with tumors harboring KRAS mutations had a similar outcome to either EGFR TKI or chemotherapy [3]. Tumors with KRAS mutations have been shown to have worse outcome compared to patients with wild type KRAS (WT) both when treated with surgery [23] or with chemotherapy [24].

The aim is to study the distribution of common and rare EGFR and KRAS mutations sent from 8 regional hospitals to the university pathology department. The quality of tumor biopsies sent in for mutational analysis was assessed and mutation status was related to treatment with EGFR TKI outcome.

## Methods

### Patients

This study concerns all the NSCLC tumor samples from eight regional Dutch hospitals during the period of November 2008 until April 2011 that were tested for mutational status by a central pathology department. Data on gender, smoking status, age at diagnosis, stage at diagnosis, localization of metastases, start date and (different) lines of treatment received were collected. Tumor samples were obtained by either bronchoscopy, transthoracic lung biopsies and/or from pulmonary resections and were sent to the respective pathology department for histological examination. Histology was according to 2004 WHO criteria [25]. Response to treatment was performed according to RECIST criteria [26].

### Sample collection procedure and DNA extraction

From each formalin-fixed and paraffin embedded (FFPE) tumor tissue block that was sent to the pathology department 4 µm sections were cut. After hematoxylin and eosin staining, slides were evaluated by an experienced lung pathologist for the presence of sufficient tumor tissue and estimating the percentage of tumor cells. Samples with clearly less than 50% tumor cells were defined as inadequate for EGFR/KRAS mutation testing. Areas with >50% tumor cells marked by the pathologist on the slide. This area was scraped from the slide using a scalpel and dissolved in TE-4 and 20 mg/ml Proteinase K (Life Technologies, Grand Island, NY, USA). DNA was extracted by incubation overnight at 55°C, followed by heating to 100°C for 5 minutes to inactivate proteinase K and centrifuged at room temperature at 13,000 rpm. The aqueous solution was directly used for PCR analysis or stored at −20°C. DNA concentration was measured on a ND1000 spectrophotometer (Nanodrop, Wilmington, DE, USA). All DNA isolates were set to 10 ng/µl in TE-4 prior to use. For quality control, genomic DNA was amplified in a multiplex PCR containing a control gene primer set resulting in products of 100, 200, 300, 400 and 600 bp according to the BIOMED-2 protocol [27]. Only DNA samples with PCR products of 300 bp and larger were used for mutation analysis. All samples were tested on DNA extracted from two independent slides (duplicates). All standard precautions were taken to avoid contamination of amplification products using separate laboratories for pre- and post-PCR handling. To avoid cross-contamination, a new microtome blade was used each time a new sample was sectioned.

Either direct sequencing or high resolution melting (HRM) with confirmatory direct sequencing was performed according to protocol. Identical mutations in forward and reverse sequencing was required before a positive result is reported. The protocol is detailed in Appendix S1. The primers used for direct sequencing or HRM are described in supplemental table 1.

**Table 1:** Patient and tumor characteristics from samples sent to central laboratory for mutation analysis.

	*N*	Percentage
Number of patients	442	100
Number of biopsies	474	
Histology		
Adenocarcinoma	353	80
SCC	27	6
Large cell undifferentiated	42	9
Adenosquamous	7	1
Carcinoid	3	1
Salivary gland	2	1
NSCLC-NOS	8	2

SCC is squamous cell lung carcinoma. NSCLC-NOS is non-small cell lung cancer – not otherwise specified.

### Informed Consent and Ethics

When patients first visited the outpatient department, written informed consent for blood and tumor tissue was obtained for mutational analysis. EGFR and KRAS tests were performed as part of routine diagnostic approach and the outcome of these tests was documented in the patient file and communicated with patients. Because this is a retrospective study to collect and analyze clinical patient data, under the Dutch Law for human medical research (WMO), no consent was necessary from the medical ethics committee. Data were coded and not traceable to the individual patient.

### Statistics

Descriptive statistics were performed for patient and tumor characteristics. Frequencies of common and rare mutations were tabulated. The frequency of EGFR and KRAS mutations were compared with available data on lung tissue from the Catalogue Of Somatic Mutations In Cancer database, (Cosmic DB; http://www.sanger.ac.uk/genetics/CGP/cosmic/). The relation between the presence or absence of mutations and the occurrence of most common tumor metastases was determined using the two sided Fisher exact test. For this particular analysis the patients with either an EGFR or a KRAS mutation were compared with patients who were scored as being both EGFR and KRAS WT. Overall survival (OS) was calculated from the date of diagnosing stage IV disease until censorship or death. Only patients with available clinical data who had progressed to stage IV disease and subsequently were treated were included for survival analysis. All patients treated with an EGFR TKI irrespective of their mutational status were evaluated for overall survival.

Univariate Cox regression analysis was performed with the covariates age, gender, histology (presence of adenocarcinoma, squamous cell and large cell carcinoma), KRAS and EGFR mutation status, metastatic site (brain, bone, lung) were also analyzed. Variables with *p*-value less than 0.20 were used for the multivariate analysis.

All statistical analysis was performed using SPSS version 18.0. Nominal *P*-values less than 0.05 were considered significant.

## Results

### EGFR and KRAS mutations

From November 2008 until April 2011 474 samples from 442 patients were sent to the central pathology department for mutation analysis. The most common histological classification was adenocarcinoma (80%), 8% of the samples came from histological subtypes not associated with EGFR mutations (Table 1).

Two hundred and twenty one patients (60.1% of all tested patients, 50% of all patients) were EGFR and KRAS WT. Thirty eight patients (10.9% of all tested patients, 8.6% of all patients) had an EGFR mutation (Table 2). In 5 patients, 2 different EGFR mutations coincided in the same tumor tissue resulting in a total of 43 mutations. Thirty of 38 patients with EGFR mutations (79%) were activating EGFR mutations. Only one patient had a T790M mutation in the primary tumor. TTF-1 positive adenocarcinomas showed an EGFR mutation more often than those who were TTF-1 negative (26/150 vs 1/50, Fisher's exact 2-sided test, *p* = 0.01).

**Table 2:** Distribution of EGFR mutations in advanced non-squamous cell lung carcinoma.

Type of EGFR mutation	Sensitivity	Frequency of mutations	Percentage %	Frequency in COSMIC^1^
p.K708N	Unknown	1	2.3	ND
p.G709_T710>M	Unknown	1	2.3	ND
p.G719	Sensitive	3	7.0	0.008
Exon 19 deletion	Sensitive	16	37.2	0.157
p.S768I	Resistant	1	2.3	<0.5%
p.V769M	Resistant	1	2.3	<0.5%
p.D770GY	Unknown	2	4.7	<0.5%
p.D770_N771>SVD	Resistant	1	2.3	<0.5%
p.T790M	Resistant	1	2.3	0.011
p.L833F	Unknown	2	4.7	<0.5%
p.A840T	Unknown	2	4.7	<0.5%
p.L858R	sensitive	11	25.6	0.145
p.L861R	sensitive	1	2.3	<0.5%
Total		43 mutations*	100	

1 From the Cosmic data base (retrieved on 05-02-2013) containing 13030 mutations in 48781 samples.

* 43 mutations were observed in 38 patients, 5 patients had double mutations.

The combination of double EGFR mutations were p.G719C, p.S768I, G719S L861R, G719C D770GY, L833F L858R and T790M L858R.

A total of 110 of patients (30% of all tested patients, 24% of all patients) had a KRAS mutation with G12C (41%) and G12V (18%) being the most frequent mutations and showing a similar distribution as in the Cosmic database (Table 3). We also found 1 (1%) rare KRAS mutation in codon 13, (p.G13Y). In addition, 2 patients had KRAS mutations outside the hotspot (p.V14L and p.L19F), these are non-functional. This means that in a total of 108 patients a functional KRAS mutation was detected in our cohort. The comparison of mutational results in the different subtypes of NSCLC in our population is shown in table 4.

**Table 3:** Distribution of codon 12/13 KRAS mutations in advanced non-squamous cell lung carcinoma from this study compared with the frequency distribution in the Cosmic database.

Mutation type	Frequency/no of pts	Percentage	Frequency in Cosmic^1^
p.G12C	45	41.7	40.5
p.G12V	20	18.5	19.7
p.G12D	17	15.7	16.7
p.G12A	11	10.2	6.4
p.G13C	5	4.6	2.9
p.G12F	4	3.7	0.7
p.G12S	2	1.9	4.3
p.G13D	2	1.9	2.5
p.G12R	1	0.9	2
p.G13Y^2^	1	0.9	ND
Total	108	100%	100%

ND =  Not Described.

^1^ From the Cosmic data base (retrieved on 05-02-2013) containing 3504 mutations in 21589 samples.

^2^ This mutation (c37_38GG>TT, p.G13Y) was detected in 2 independent non-synchronous biopsies of the same patient.

Two KRAS mutations (p.V14L (not present at Cosmic) and p.L19F 2/2742 (present at Cosmic retrieved on 05-02-2013) were found outside codon 12/13 (considered as non-functional).

**Table 4:** Distribution of EGFR and KRAS mutations and their wild types in histological NSCLC subtypes of 442 patients.

	EGFR mutation	%	KRAS mutation	%	EGFR/KRAS WT	%	Insufficient material	%	Total
Adenocarcinoma	33	9.3	98	27.8	164	46.5	58	16.4	353
Squamous cell carcinoma	0	0	2	7.4	21	77.8	4	14.8	27
Adenosquamous	1	14.3	1	14.3	4	57.1	1	14.3	7
NSCLC NOS	3	5.4	9	16.4	32	58.2	11	20	55
Total	37*	8	110**	25	221	50	74	17	442

* Not including a patient with dual EGFR/KRAS mutation.

** Including 2 patient with a non-functional KRAS mutation and 1 patient with a dual EGFR/KRAS mutation.

### Quality of tumor samples for mutation analysis

**Figure 1:**
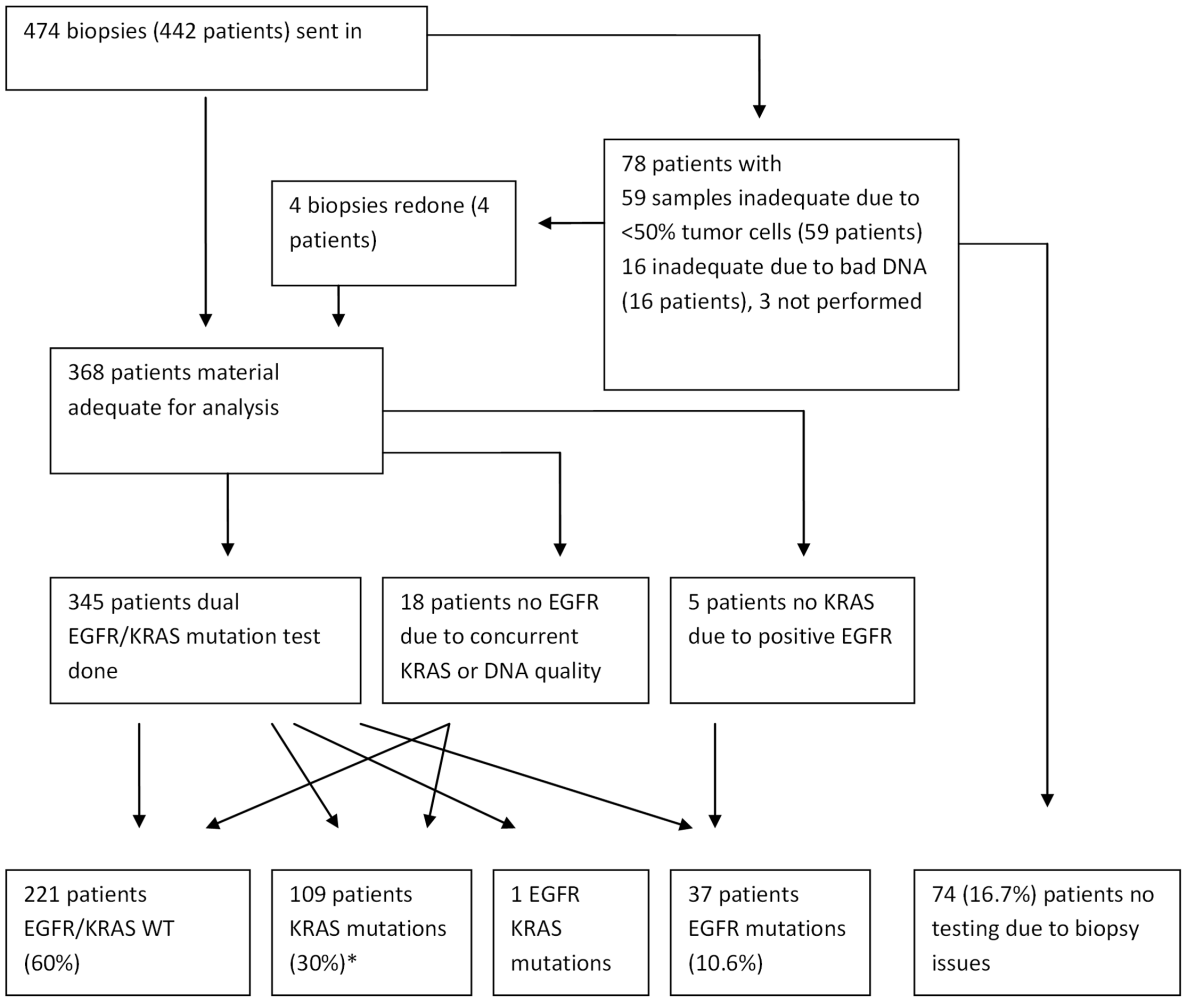
Flow chart for biopsy specimens sent in and result of mutation analysis. * 2 KRAS mutations are outside of the hotspot, these are probably non functional.

Seventy five tumor samples ((16%) were not adequate for mutation analysis. In 59 samples tissue contained less than 50% tumor cells (mostly because of extensive intermingling inflammation) and in 16 the quality of DNA appeared unsuitable for mutation testing. In 4 of these patients an adequate tissue sample was yielded by re-biopsy. In 3 tumors no further mutation analysis was performed (SCC/carcinoid). This means that from 74(75+3–4) (17%) patients no results were obtained from mutational analysis. In a total of 345 patients the tumor samples were adequate for both EGFR and KRAS analysis. A single KRAS or EGFR mutation analysis was performed in the tumor samples of 18 and 5 patients, respectively (Figure 1).

### EGFR and KRAS mutations and metastases distribution

Using the clinical data from 303 patients, we were able to analyze the preference for the known common metastatic regions for the patients with NSCLC with KRAS and EGFR mutational status. Pulmonary nodules *(p* = 0.01), vertebral (*p* = 0.03) and other bone metastasis (*p* = 0.04) were identified to be significantly associated with EGFR mutations. No association was found between EGFR mutations and pleural (*p* = 0.15), cerebral (*p* = 1.0), hepatic (*p* = 0.46) or adrenal (*p* = 0.37) metastatic localizations. None of these sites were associated with KRAS mutations.

### Survival analysis

In univariate analysis from the clinical data, large cell histology (HR 1.8, 95% CI., 1.2–2.8, *p*<0.01) and spinal bone metastasis (HR 1.5, 95% CI., 1.0–2.2, *p* = 0.05) were associated with a worse survival while EGFR mutation (HR 0.4, 95% CI., 0.2–0.7, *p*<0.01) was associated with a better survival. In a multivariate model, histology (large cell carcinoma, HR 2.2, 95% CI., 1.4–3.4, *p*<0.01), spinal bone metastasis (HR 1.7, 95% CI., 1.2–2.6, *p*<0.01), and mutational status (EGFR mutation, HR 0.3, 95% CI., 0.1–0.6 *p*<0.01) were significantly associated with survival. (Table 5).

**Table 5:** Univariate and multivariate hazards ratios for overall survival in 248 patients with metastatic non-small cell lung cancer.

	Univariate			Multivariate		
Variables	HR	95% CI	*P*	*HR*	95% CI	*P*
*Histology*						
Adeno	1			1		
Squamous	1.2	0.7–2.1	0.41	1.2	0.7–2.1	0.48
Large Cell	1.8	1.2–2.8	<0.01	2.2	1.4–3.4	<0.01
*Mutation result*						
EGFR/ KRAS WT	1			1		
EGFR mutation	0.4	0.2–0.7	<0.01	0.3	0.1–0.6	<0.01
KRAS mutation	1.1	0.7–1.5	0.70	1.1	0.8–1.8	0.34
No test performed	1.2	0.8–1.9	0.33	1.4	0.8–2.1	0.31
*Metastasis* *						
Spinal bone	1.5	1.0–2.2	0.05	1.7	1.2–2.6	<0.01
Brain	0.9	0.6–1.4	0.67			
Lung	1.1	0.8–1.5	0.64			

HR>1 means a shorter survival.

* denotes presence of metastasis at specific site.

**Figure 2:**
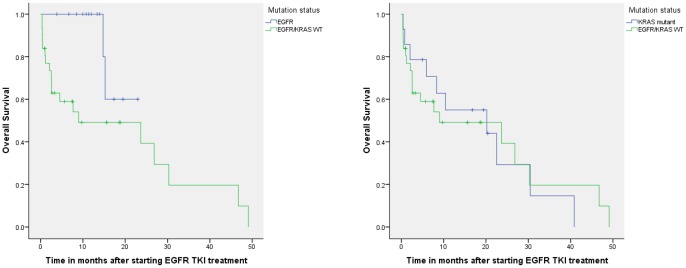
A: Overall survival in patients with non-small cell lung cancer treated with EGFR-TKI in the first and second line with or without an EGFR mutation. The median overall survival for patients with EGFR mutations (n = 14) was not reached, in patients with EGFR/KRAS WT it was 9 months (95% CI., 0–28 months, n = 31). 2B: Overall survival in patients with non-small cell lung cancer treated with EGFR-TKI in the first and second line with or without KRAS mutation. The median overall survival for patients with KRAS mutations was 20 months (95% CI., 0–46, n = 14), in patients with EGFR/KRAS WT it was 9 months (95% CI., 0–28 months, n = 31).

When selecting patients who received EGFR TKI treatment in the first or second line, the median overall survival after start of this treatment was not reached in patients with EGFR mutation (n = 14), 20 months (95% CI., 0–46, n = 14) for patients with KRAS mutation, and 9 months (95% CI., 0–28, n = 31) for patients with EGFR/KRAS WT. (Figure 2A and 2B).

### Rare EGFR and KRAS mutations and response to treatment

Mutations that were not previously described in COSMIC DB are described in table 6. Treatment with an EGFR TKI in patients with these rare EGFR mutations did not result in clinical benefit except in one patient who also had an additional activating EGFR mutation.

**Table 6:** Rare EGFR and KRAS mutations and tumor response to EGFR TKI.

Mutations	N	Response	Response	Published response to
		to chemotherapy	to EGFR TKI	EGFR TKI
*EGFR mutations*				
p.K708N	1	PD	PD (E)	[47]PR with
				gefitinib with
				p.K708M
				
p.V769M	1	PR	PD (E)	[33], [48];
				No treatment
				information
				
p. D770GY	1	PD	PR (E)	[36], [37]
with a secondary				No treatment
p.G719C mutation				information
				
				
p.D770GY;	1	PR	PD (G)	[36], [37]
without secondary				No treatment
mutation				information
				
p.L833F	1	PR	PD (G)	ND
(dual KRAS mutation)				
				
p.A840T	2	PR/PR	PD (E)/–	ND
*KRAS mutations*				
p.G13Y	1	PD	–	ND
				
p.V14L	1	PR	–	ND

PR is partial response, PD is progressive disease, –  =  no EGFR TKI treatment; (E) = erlotinib, (G) = gefitinib, ND = Not described.

## Discussion

EGFR is a cell surface protein that leads to activation of proliferation and invasion via different signal transduction pathways [28]. KRAS is a downstream target of EGFR. Activating or sensitizing mutations cause a constitutive activation of the tyrosine kinase domain of the EGFR protein, by destabilizing the autoinhibiting conformation [29]. EGFR TKI such as gefitinib have increased binding abilities for these mutant proteins. The ratios of this increased binding ability is up to 100 fold compared to wild-type EGFR protein [29].

The two most common sensitizing EGFR mutations to EGFR TKI, in frame deletions of exon 19 and the L858R mutation, [19], [30], [31], [32], [33] represented over half of all EGFR mutation patients. Other sensitizing aberrations were found in three patients having a G719X mutation and in another patient a L861R mutation [33], [34], [35]. We observed 5 rare or previously undescribed mutations (Table 2) and have characterized their response to TKI treatment (Table 6). Of specific note is the p.D770GY mutation, which was found in two patients, with different response. The first of these patients had a combination of p.D770GY and a p.G719C mutation while the second had only a p.D770GY mutation. The first patient responded to EGFR TKI and remains disease free after 15 months while the patient without the secondary mutation had progressive disease diagnosed at 4 weeks. Previously 2 cases of this mutation were described without information on tumor response [36], [37]. Our data suggest that the p.G770GY mutation does not provide benefit for EGFR TKI treatment. Furthermore, we demonstrated that also patients with one of the other 4 rare EGFR mutations (p.K708N, p.G709_T710>M, p.L833F and p.A840T) had no benefit from EGFR-TKI.

Small tumor samples mainly from bronchoscopic or transthoracic core biopsies may be a problem for adequate mutation testing. We identified causes why mutational analysis at our lab was not possible in 17% of patients. This was either due to insufficient number of tumor cells (12%) or due to insufficient DNA quality (4%) highlighting the need for adequate tumor tissue selection for mutational analysis. Retrospective studies in which long- term archived paraffin embedded tissue was used to determine EGFR status showed a low proportion of adequate tumor tissue available [1], [2], [3], [4], [5], [6]. One way to obtain more tumor cells is by repeated biopsies or cryobiopsies [38]. New technological developments are far more sensitive than previously, allowing fewer tumor cells both qualitatively (%) and quantitatively (absolute number) required for detecting mutations. However, regarding tumor heterogeneity, this increased sensitivity harbors an increased risk of sampling errors and detection of minor clones that may be less relevant for therapy. A study showed that about two thirds of all somatic mutations seemed not to be detectable across every tumor region [39].

EGFR mutations occurred most often in TTF-1 positive adenocarcinoma. Two recent studies showed this cell lineage association [40], [41]. Functionally, TTF-1 induced ROR-1 is necessary to sustain the EGFR signaling pathway in lung adenocarcinoma cell lines [42].

We identified the preference of EGFR mutant tumors to spread to intrapulmonary and to both the vertebra and other bone localizations. This contrasts with a study by Doebele et al, who observed only a preference for hepatic metastatic spread in EGFR mutant tumors [43].In contrast, we observed the typical miliary pattern of tumors with EGFR exon 19 deletion as described previously [44]. Our results for KRAS mutant tumors (71 patients) were as described previously by Doebele et al (49 patients) [43].

In our population the outcome of patients with a KRAS mutation responded similarly to KRAS WT both with respect to chemotherapy and to EGFR TKI. Previously it was demonstrated that patients with KRAS wild type have a better outcome than patients with KRAS mutations when treated with an EGFR TKI [22]. Other studies showed the presence of KRAS mutations in lung cancer to be indicative of worse outcome regardless of the treatment they received [45], [46]. In the TITAN study, there was some evidence for a higher risk of death in KRAS mutant tumor patients treated with erlotinib compared to chemotherapy but there was no elevated risk of tumor progression [4]. In our study, we did not pool the EGFR mutation positive patients with the EGFR/KRAS WT when comparing these patients with KRAS mutant patients. As patients with EGFR mutations tend to have better outcomes then EGFR WT patients, this could explain our results.

In conclusion, we found in 10.9% and 30% of all the tested patients an EGFR or KRAS mutation, respectively. We also identified 5 novel or rare EGFR mutations and 2 novel KRAS mutations in our population. Seventeen percent of patients had inadequate tumor tissue to perform mutation analysis, mostly due to insufficient tumor volume and/or percentage. There was no difference in overall survival after starting EGFR-TKI in patients with KRAS mutation and EGFR/ KRAS WT.

## Supporting Information

Appendix S1 (DOC)
