# Supplementary material for: Common and Rare EGFR and KRAS Mutations in a Dutch Non-Small-Cell Lung Cancer Population and Their Clinical Outcome
Source: PLoS One. 2013 Jul 29;8(7):e70346. doi: 10.1371/journal.pone.0070346 (PMC3726644; doi:10.1371/journal.pone.0070346)
Supplement: Appendix S1 — (DOC) [file pone.0070346.s001.doc]

Supplementary appendix 1: information on mutation testing

**Direct sequence analysis for the detection of mutations in exons 18-21 of the EGFR gene**

For the detection of mutations in exon 18-21 of the EGFR gene, 100 ng genomic DNA was analyzed by PCR using 12 specific primers covering exons 18-21 (EGFR: CCDS5541.1) (supplementary Table 1) followed by direct bidirectional Sanger sequencing. Briefly, the PCR reaction mixtures with a final volume of 50 μl contained 0,2 µM forward (F) primer, 0,2 µM reverse (R) primer, 1× reaction buffer (PEII, Life Technologies), 1.5 mM MgCl2, 0,2 mM dNTP and 1 units of Taq DNA polymerase (Life Technologies) The PCR was performed using the following conditions; 95 °C/10 min, 40 cycles 94 °C/30 sec, 58°C/60 sec, 72 °C/1 min, and 72 °C for 10 min. PCR products were checked by electrophoresis of 10 μl of each amplified sample on a 1,5% agarose gel. To purify and concentrate amplified DNA, a High Pure PCR Product Purification Kit (Roche, Mannheim, Germany) was used before diluting the DNA samples. Direct bi-directional sequence analysis of PCR products (in duplicate) was performed using the BigDye Terminator v3.1 Cycle sequencing kit (Applied Biosystems, Foster City, CA, USA) using each of the same primers used for PCR and the ABI PRISM 310 Genetic Analyzer (Life Technologies) performed by Baseclear (Leiden, the Netherlands, www.baseclear.com). All sequences were read in forward and reverse direction. Both reads were analysed with CLC Genomics Workbench ([www.clcbio.com](http://www.clcbio.com/)). Using serial dilutions of DNA from mutant FFPE samples of clinical anonymous specimens previously identified with EGFR L858R and EGFR exon-19del mixed in a background of WT tonsillar PPFE DNA, the analytical sensitivity to detect EGFR mutations in FFPE samples was 48%.

**High resolution melting (HRM) PCR for the detection of mutations in exon 2 of the KRAS gene with direct sequencing confirmation**

For detecting mutations in exon 2 (including codon 12/13) of the KRAS gene (CCDS8702.1), DNA samples were first analyzed using high resolution melting (HRM) and conformation of the mutation in the HRM-positive cases using direct sequence analysis as reported previously. [1] The KRAS exon 2 primers and probes are shown in supplementary Table 1. The PCR for the HRM analysis was performed on a LightCycler 480 (Roche). PCR reaction mixtures with a final volume of 20 μl contained 500 nM forward primer, 500 nM probe with a 3’-conjugated C3 spacer, 100nM reverse primer (all primers/probes were purchased from IDT, Leuven) and 10ng DNA in 1x HRM Mastermix (Roche). Two dilutions (10% and 20% mutant DNA isolated from human colon cancer cell line Lovo (ATCC: CCL-229 with homozygous p.G13D mutation) were used as positive controls in each experiment. The cycling and melting conditions were as follows: one cycle of 95◦C/2 min; 50 cycles of 95◦C/30 s; 64◦C/30 s; 72◦C/30 s; and one cycle of 72◦C/60s, 95◦C/20s, 55◦C/20s with a final melting in two steps: Melt 1 (55–78 ◦C at 0.06 ◦C/s) followed by Melt 2 (78-95 ◦C, 0.06 ◦C/s) and continuously recording of the fluorescent level. The change of fluorescence is converted to a melting peak by plotting the negative derivative of the fluorescent signal corresponding to the temperature (−dF/dT) on the LightCycler480 software. KRAS mutations are identified by the comparison of each sample’s peak plot with the WT peak (a mixture of 5 independent FFPE DNA samples isolated from human tonsils) and mutant (DNA isolated from human material) reference DNA. Normalized and temperature-shifted difference plots of the LightCycler480 software release 1.5 and LightCycler 480 Gene Scanning Software (Roche, Mannheim, Germany) were additionally used to aid mutation identification. The HRM assay was performed in duplicate on each sample. Using serial dilutions of DNA from mutant FFPE samples of 3 colon carcinomas (with KRAS p.G12A and p.G12D mutation) in a background of WT tonsil PPFE DNA, the analytical sensitivity of KRAS HRM in FFPE samples was determined at 20%. The original genomic DNA of cases with an abnormal HRM melting curve, characteristic for the presence of a mutation, was subjected to direct bidirectional Sanger sequence analysis to confirm the specific KRAS mutation as described for EGFR mutation detection using specific sequence primers (Table 1). The analytical specificity of the KRAS HRM assay was validated on a series of 208 FFPE sample of clinical colon carcinomas compared to direct sequence analysis (96%). Sequence analysis of the HRM product using M13-sequencing-primer was only performed in case no nucleotide changes were observed in the sequence derived from the new PCR of the original genomic DNA.

Supplementary Table 1: Primers used for sequencing EGFR and KRAS genes.

Primer sequence EGFR PCR

EGFR exon 18 F 5’-GCT GAG GTG ACC CTT GTC TC-3’

EGFR exon 18 R 5’-CTC CCC ACC AGA CCA TGA-3’

EGFR exon 19 F 5’-CAT GTG GCA CCA TCT CAC A-3’

EGFR exon 19 R 5’-CAG CTG CCA GAC ATG AGA AA-3’

EGFR exon 20 F 5’-CAT GCG TCT TCA CCT GGA A-3’

EGFR exon 20 R 5’-AGC AGG TAC TGG GAG CCA AT-3’

EGFR exon 21A F 5’-GAA TTC GGA TGC AGA GCT TC-3’

EGFR exon 21A R 5’-TGC CTC CTT CTG CAT GGT AT-3’

EGFR exon 21B F 5’-GAG GAC CGT CGC TTG GTG-3’

EGFR exon 21B R 5’-ATC CTC CCC TGC ATG TGT TA-3’

Primer sequence KRAS PCR

KRAS Fex 5’- GTTCTAATATAGTCACATTT -3’

KRAS Rex 5’- ACTCATGAAAATGGTCAGAGAAACCTTTAT -3’

HRM Primers and probes of exon 2 of the KRAS gene

KRAS HRM-exon 2 F 5’-GTA AAA CGA CGG CCA GTC ACA TTT TCA TTA TTT TTA TTA TAA GGC -3’

KRAS HRM-exon 2 R 5’-GAT TCT GAA TTA GCT GTA TCG TCA AG-3’

KRAS HRM-exon 2 WT probe 5’-CTT GCC TAC GCC ACC AGC TCC AAC T-3’

References

1. Kramer D, Thunnissen FB, Gallegos-Ruiz MI, Smit EF, Postmus PE, et al. (2009) A fast, sensitive and accurate high resolution melting (HRM) technology-based assay to screen for common K-ras mutations. Cell Oncol 31: 161-167.
